# Protective Effects of the Flavonoid Chrysin against Methylmercury-Induced Genotoxicity and Alterations of Antioxidant Status, *In Vivo*

**DOI:** 10.1155/2015/602360

**Published:** 2015-02-24

**Authors:** Eduardo Scandinari Manzolli, Juliana Mara Serpeloni, Denise Grotto, Jairo Kennup Bastos, Lusânia Maria Greggi Antunes, Fernando Barbosa, Gustavo Rafael Mazzaron Barcelos

**Affiliations:** ^1^Department of Clinical Analyses, Toxicology and Food Sciences, School of Pharmaceutical Sciences of Ribeirão Preto, University of São Paulo, Avenida do Café s/n°, 14040-903 Ribeirão Preto, SP, Brazil; ^2^Department of Pharmaceutical Sciences, School of Pharmaceutical Sciences of Ribeirão Preto, University of São Paulo, Avenida do Café s/n°, 14040-903 Ribeirão Preto, SP, Brazil

## Abstract

The use of phytochemicals has been widely used as inexpensive approach for prevention of diseases related to oxidative damage due to its antioxidant properties. One of dietary flavonoids is chrysin (CR), found mainly in passion fruit, honey, and propolis. Methylmercury (MeHg) is a toxic metal whose main toxic mechanism is oxidative damage. Thus, the study aimed to evaluate the antioxidant effects of CR against oxidative damage induced by MeHg in Wistar rats. Animals were treated with MeHg (30 *µ*g/kg/bw) in presence and absence of CR (0.10, 1.0, and 10 mg/kg/bw) by gavage for 45 days. Glutathione (GSH) in blood was quantified spectrophotometrically and for monitoring of DNA damage, comet assay was used in leukocytes and hepatocytes. MeHg led to a significant increase in the formation of comets; when the animals were exposed to the metal in the presence of CR, higher concentrations of CR showed protective effects. Moreover, exposure to MeHg decreased the levels of GSH and GSH levels were restored in the animals that received CR plus MeHg. Taken together the findings of the present work indicate that consumption of flavonoids such as CR may protect humans against the adverse health effects caused by MeHg.

## 1. Introduction

The use of phytochemicals (PhC) has been proposed as inexpensive approach for prevention of diseases associated with oxidative stress, such as cardiovascular disorders, degenerative diseases, and cancer, due to its antioxidant properties [1–3]. PhC are bioactive compounds, nonnutrients found in fruits, vegetables, grains, and other plants that are associated with a decreased risk to develop several diseases [1]. In this context, it is notable that several studies have been carried out aiming to assess the potential protective effects of PhC in many laboratory models, showing very promising results [3–7].

The flavonoid chrysin (CR; 5,7-dihydroxy-2-phenyl-4H-chromen-4-one) is a natural and biologically active compound; it is found mainly in honey, passion fruit (*Passiflora *sp.), and propolis [8–10]. Previous studies were already performed aiming to determine the concentrations of CR in honey and propolis; for example, Lachman et al. [11] assessed the CR content in several types of honeys and found values ranging from 0.10 mg/kgg (honeydew honeys) to 5.3 mg/kg (forest honeys); in another study, Siess et al. [12] found levels of CR in propolis as high as 28 g/L, demonstrating that honey and propolis are a rich source of this flavonoid.

Compared with other flavonoids, few studies have been carried out in order to determine the beneficial effects of CR. Some of these investigations yielded promising results. For example, Uhl et al. [13] demonstrated antimutagenic properties against benzo(a)pyrene B(a)P induced DNA-damage in HepG2 cells and* Salmonella typhimurium*; Anand et al. [14] showed that CR reduces the disturbances of redox status, named superoxide dismutase (SOD), catalase (CAT), glutathione peroxidase (GPx), and glutathione (GSH) in liver, kidney, and brain tissues of rats treated with D-galactose.

Fish is considered a healthy food since it is a good source of proteins, is poor in saturated fats, and has high amounts of polyunsaturated fat acids, which may protect against cardiovascular disorders; and populations that traditionally consume large amounts of fish generally show low mortality rates from coronary diseases [15, 16]. On the other hand, fish consumption is also an important source of human exposure to a variety of bioactive toxicants such as methylmercury (MeHg) and arsenic (As) [17–19] which may interact with the health effects of fish intake [20–22]. It is well established that chronic exposure to MeHg is associated with neurological disorders [23–25] and with adverse effects on the cardiovascular system [26, 27]. One of the main mechanisms responsible for MeHg toxicity is the induction of oxidative stress. Earlier data have consistently shown that MeHg exposure can lead to disturbances in the redox status, causing oxidative damage in macromolecules, such as lipids and DNA [28, 29].

Despite the low number of studies concerned with evaluating the protective effects of PhC against MeHg-induced adverse effects, the aim of present study was to investigate potential protective effects of CR against the toxic effects caused by MeHg, through monitoring of DNA-damage by comet assays in peripheral blood cells and hepatocytes and by determination of GSH levels in blood of Wistar rats.

## 2. Materials and Methods

### 2.1. Chemicals

Methylmercury chloride (CAS 115-09-3), reduced glutathione (GSH, CAS 70-18-8), glutathione reductase (GR, CAS 9001-48-3), sodium azide (CAS 26628-22-8), trypan blue (CAS 72-57-1), ethidium bromide (CAS 1239-45-8), and 5-5′-dithiobis-(2-nitrobenzoic acid) (DTNB; CAS 69-78-3) came from Sigma-Aldrich (St. Louis, MO, USA). CR (CAS 480-40-8) was kindly provided by Professor Dr. Jairo K. Bastos from School of Pharmaceutical Sciences of Ribeirão Preto, São Paulo, Brazil. The purity of CR used in the experiments was ≥95%. Ketamine and xylazine were from Bayer (São Paulo, Brazil). Low melting point agarose (LMP) and normal melting point (NMP) agarose were obtained from Invitrogen (California, CA, USA). All other chemicals, reagents, and buffers were analytical grade products from Sigma (St. Louis, MO, USA).

### 2.2. Animals

The experiments were carried out with 2-month-old male Wistar rats, weighing on average 200 ± 20 g, which were obtained from Central Animal House (University of São Paulo, Ribeirão Preto, Brazil). The animals were kept under a 12 h light/dark cycle in an acclimatized room at 22–25°C and had free access to food (standard ration from Guabi, Campinas, Brazil) and water. The animals were used according to the guidelines of the Committee on Care and Use of Experimental Animal Resources, University of São Paulo, Brazil (approved protocol number 09.1.457.53.1).

### 2.3. Experimental Design

The dose of MeHg (30 *μ*g/kg of body weight (bw)) was chosen on the basis of previous studies of our group which showed consistently that this concentration is able to induce DNA damage and disturbances of redox status and also reflects the exposure levels of individuals from Amazonian region [30–32] and from other regions which also have high levels of MeHg exposure [33]. Treatments with CR were chosen according to previous articles [14, 34].

The animals were divided in eight groups (six animals/group): (I) control (mineral oil); (II) MeHg (30 *μ*g/kg bw); (III) CR I (0.10 mg/kg bw); (IV) CR II (1.0 mg/kg bw); (V) CR III (10 mg/kg bw); (VI) CR I + MeHg; (VII) CR II + MeHg; and (VIII) CR III + MeHg; the animals were treated daily by gavage for a period of 45 days. After the treatment, the rats were killed by an overdose of ketamine and xylazine (300 and 30.0 mg/kg bw, resp.). Subsequently, blood was collected by decapitation and used for comet analyses and to determine the GSH levels. The livers were rinsed with phosphate buffered saline (PBS, pH 7.4) before removal.

### 2.4. Comet Assays with Peripheral Leukocytes and Hepatocytes

Whole blood was used for the determination of DNA damage in leukocytes [35]. Samples of liver were collected after euthanasia, and 0.20 g of each organ was placed in 1.0 mL of chilled Hank's balanced salt solution in a Petri dish, sliced into fragments with a pair of scissors, and filtered through two layers of gauze [36].

The SCGE assays were carried out according to the protocol of Singh et al. [37]. Briefly, 20 *μ*L of blood or nuclei from liver suspensions was transferred to agarose-coated slides which were coverslipped and cooled at 4°C for 20 min. After removal of the coverslips, the slides were immersed in fresh lysis solution for 1 h at 4°C. Thereafter, they were transferred to an electrophoresis chamber with buffer (300 mM NaOH and 1.0 mM EDTA pH > 13) and electrophoresis was conducted under standard conditions (25 V; 300 mA; 1.25 V/cm) for 20 min. Subsequently, the slides were neutralized, air-dried, and fixed in absolute ethanol for 10 minutes; then they were stained with ethidium bromide and evaluated with a fluorescence microscope (Nikon, Japan) under 40x magnification. From each sample, two slides were made and from each, 50 cells were evaluated per animal. Comets were scored using the Comet Score software from Tritek (Sumerduck, VA, USA); the percentage of DNA in tail was determined as a parameter of DNA damage. All experiments were carried out according to the guidelines for SCGE assays [36]. The trypan dye exclusion method [38] was used to determine cell viability immediately before the comet assays and 300 cells were evaluated per group. In all treatments, the viability was higher than 80%.

### 2.5. Total Thiols (GSH) Levels

Total thiols (taken here as GSH) were determined in erythrocytes by addition of DTNB as described by Ellman [39]. DTNB, a symmetric aryl disulfide, reacts with free thiols to form disulfide plus 2-nitro-5-thiobenzoic acid. The reaction product was quantified by measurement of absorbance at 412 nm with a spectrophotometer (Micronal B380 UV–Vis, São Paulo, Brazil). Results are expressed as micromoles per milliliter (*μ*mol/mL) in blood.

### 2.6. Statistical Analysis

All data analyses were performed with the GraphPad Prism version 6.01 for Windows (La Jolla, CA, USA). Results are reported as means ± standard deviations (SD). The results of different experiments were analyzed using one-way ANOVA and Dunnett's test. *P* values ≤ 0.050 were considered as statistically significant.

## 3. Results

**Figure 1:**
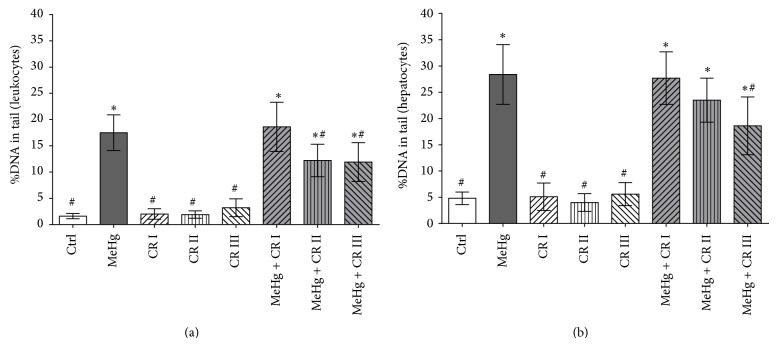
Impact of oral treatment of rats with CR on induction of DNA-damage by MeHg in (a) lymphocytes and (b) hepatocytes. The animals were treated by gavage with different doses of the flavonoid (CR I: 0.1 mg/kg/bw/day; CR II: 1.0 mg/kg/bw/day; and CR III 10 mg/kg/bw/day) in combination with the metal (30 *μ*g/kg/bw/day) over a period of 45 days. Bars indicate means ± SD of results obtained with six animals per group. Stars indicate significant difference from negative control group; hashes indicate significant difference in comparison to the MeHg group (*P* ≤ 0.050; one-way ANOVA and Dunnett's test).

The results of the experiments concerning the impact of CR on MeHg-induced comet formation are summarized in Figure 1. Exposure of the animals to the metal compound increased the extent of DNA-migration in leukocytes and liver cells 9.3 and 4.9-fold over the background values while all treatments with CR did not cause significant DNA-damage under our experimental conditions. Comet formation was significantly reduced when the flavonoid was administered in combination with the metal. In leukocytes, the decrease of comet formation at the highest doses (1.0 and 10 mg/mL) was 33 and 35%, respectively, while in hepatocytes only the highest dose was able to reduce MeHg induced DNA-damage.

**Figure 2:**
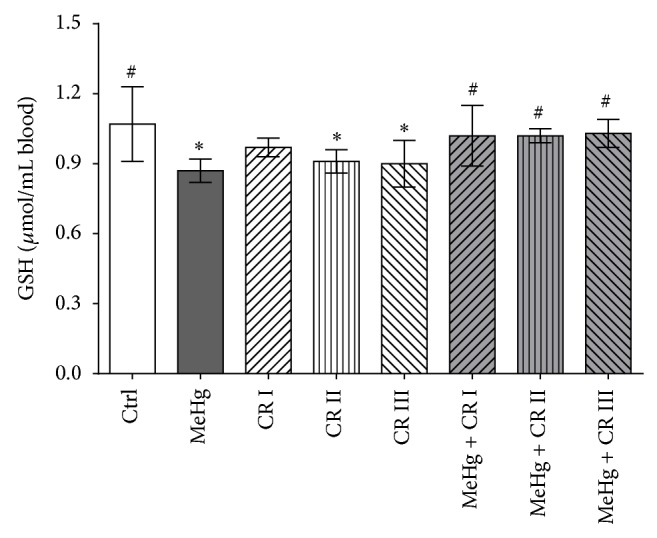
Impact of oral treatment of rats with CR on the levels of GSH in blood. The animals were treated by gavage with different doses of the flavonoid (CR I: 0.1 mg/kg/bw/day; CR II: 1.0 mg/kg/bw/day; and CR III 10 mg/kg/bw/day) in combination with the metal (30 *μ*g/kg/bw/day) over a period of 45 days. Bars indicate means ± SD of results obtained with six animals per group. Stars indicate significant difference from negative control group; hashes indicate significant difference in comparison to the MeHg group (*P* ≤ 0.050; one-way ANOVA and Dunnett's test).

The results of the measurements of the GSH levels in blood are summarized in Figure 2. Treatment with the metal reduced the levels of GSH when compared to negative controls. Furthermore, it can be seen that treatment with the higher doses of CR had a clear impact on the GSH concentrations and reduced the GSH concentrations by 15 and 16%, respectively.

Figure 2 also shows the levels of the tripeptide GSH which were measured after combined treatment of the animals with MeHg and different doses of the flavonoid. When the animals were exposed simultaneously to the metal compound and to CR, the GSH concentrations were restored to those found in untreated control animals.

## 4. Discussion 

Taken together, the findings of the present work indicate that consumption of flavonoids such as CR may protect humans against the adverse health effects caused by exposure to MeHg.

The observation of comet formation in white blood cells and hepatocytes of the animals after treatment with MeHg is in agreement with results of earlier studies [31, 32, 40]. In addition, we demonstrated previously that MeHg increases the formation of 8-hydroxy-2′-deoxyguanosine in HepG2 cells [5]. Jin et al. [41] also observed increase of this parameter in rats that were exposed to the metal compound. These findings give pieces of evidence that oxidative damage accounts for the comet formation which we observed in the present study.

*In vitro *results concerning the genotoxic properties of CR are, so far, contradictory and are related to the models that were used. For example, Resende et al. [42] showed that concentrations between 14.3 and 174.7 nmol/plate were not able to induce mutagenic effects in* Salmonella typhimurium *TA 98 and TA 100 with or without S9 fraction. On the other hand, Uhl et al. [13] demonstrated that CR induces MN formation in HepG2 cells in doses ranging from 15 to 35 *μ*g/mL; also, Oliveira et al. [43] reported increase of MN induction in HepG2 cells exposed to the flavonoid (1.0–15 *μ*M). In the same work, the later authors found that high doses of CR are able to induce mutagenic effects in* S. typhimurium *TA 98 and TA 100 (with or without S9 fraction). Resende et al. [42] reported that higher concentrations of CR (116.4 and 174.7 nmol/plate) induce significant number of revertants per plate in* S. typhimurium* TA 102. In this context, it is notable that flavonoids and other phytochemicals may act as prooxidants at high concentrations [44, 45].

Despite the large number of* in vitro* studies which aimed to assess the genotoxicity of CR, to our knowledge, there are no* in vivo *studies and there are no works that evaluated the potential genotoxic effects of CR on animals in subchronic treatments as the present study. Here, we observed that CR was not able to induce comet formation in all doses tested. This difference between the observations in mammal cells culture, such as HepG2, and the present data may be explained, at least partly, to the metabolism system of them. HepG2 cells possess mainly high expression of enzymes of phase I of metabolism [46]; the same occurs in systems that used the S9 fraction (which also have high levels of phase I enzymes) [47], while in animals, the metabolism comprises a balanced expression of phase I and phase II enzymes [48].

It is well documented in* in vitro* and* in vivo* experiments that MeHg exposure leads to formation of reactive species that may cause oxidative damage of macromolecules [28, 29]. Furthermore, it was also shown that the MeHg binds to endogenous biomolecules with –SH groups; this observation explains the decrease of the GSH levels which was seen in the present experiments and is in accordance with previous studies [49, 50].

One of the most important mechanisms to explain the oxidative damage induced by Hg exposure is its high reactivity with sulhydryl groups (−SH) of macromolecules, which may inactivate them [51]. GSH is the main intracellular nonprotein free thiol, and it is also one of the most important antioxidants in the body [52]. It is conceived that GSH play a role as a first cellular defense against Hg compounds. The metal compounds bind to GSH covalently, through cysteine residues and thus, its deleterious effects are minimized. This protective effect mediated by GSH, however, decreases its concentrations, and then the cells may be more susceptible to oxidative damage through the accumulation of reactive oxygen species (ROS) normally neutralized by GSH [53, 54].

The reactive species then attack proteins, DNA and lipids [55], inducing oxidative damage. Hg interferes with the activity of several antioxidant enzymes; for example, the activity of superoxide dismutase (SOD), catalase (CAT), and glutathione peroxidase (GPx). However, so far, there is no general consensus among the data; that is, some studies reported increase of activity of certain antioxidant enzymes while others showed a reduction in activity of these enzymes. Ariza et al. [40] demonstrated that HgCl_2_, inducing the formation of H_2_O_2_, stimulates SOD activity and does not affect the activity of CAT and GPx, whereas Hussain et al. [56] showed an increase in the activity of GPx and CAT. In general, acute exposure to Hg leads to decrease in enzymatic activity; on the other hand, exposure to prolonged periods seems to not change the enzyme patterns, possibly due to an indirect compensatory response of the cells to increased oxidative stress, such as self-protection mechanism [49].

We also showed that the higher doses of CR (1.0 and 10 mg/kg bw, resp.) reduce the levels of GSH when compared to negative controls. A possible explanation for this observation is that antioxidant properties of CR may modulate a feedback mechanism on the antioxidant system triggered by administration of the flavonoid to the animals, which was already seen in a previous study where female rats were exposed to several flavonoids, including CR [57].

The expectation that CR prevents the adverse effects of MeHg is based on results of previous studies which indicate that the flavonoid shows DNA-protective effects against radiation and chemically induced DNA-instability. For example, Benković et al. [58] showed that CR at dose of 100 mg/kg bw was able to decrease the comet formation in leukocytes of gamma-irradiated mice; in a further study of the same authors [59] it was observed that pretreatment of CR protects against DNA-damage induced by gamma radiation in human lymphocytes cell cultures. Uhl and coworkers [13] observed that CR decreases MN formation induced by B(a)P and 2-amino-1-methyl-6-phenylimidazo[4,5-*b*]pyridine exposure. In the same study, the authors also observed protective effects in* S. typhimurium *strains TA 98 and TA 100 against B(a)P. A recent study of Resende et al. [60] gives further pieces of evidence concerning the antigenotoxic effects of CR against several indirect and direct mutagens (4-nitro-o-phenylenediamine, sodium azide, mitomycin C, B(a)P, aflatoxin B1 and 2-anthramine) in* S. typhimurium* TA 98, 100, and 102, with or without activation mix (S9). Another hypothesis is that CR can bind directly to MeHg, chelating the metal, and prevent the direct oxidative damage induced by exposure to the metal, which was already reported by several studies (for a comprehensive review, see Flora 2009; 2010) [61, 62]. These previous findings give further support that flavonoids may act not only as direct antioxidant, inactivating free radicals, but also as an attractive tool for prevention of adverse health effects induced by heavy metals by use of chelation therapy.

Finally, we also observed that CR ameliorated the disturbances in the levels of GSH induced by MeHg exposure; in line with our findings, several studies consistently showed the antioxidant properties of CR. Ciftci et al. [63, 64] observed that the flavonoid was able to reduce the alterations on the antioxidant enzymes SOD, CAT, and GPx and on the levels of GSH in kidneys and livers of mice treated with 2,3,7,8-tetrachlorodibenzo-*p*-dioxin. In another study, Siess et al. [12] showed that CR reduces the disturbances of the redox status in liver, kidney, and brain tissues of rats which were induced with D-galactose.

As mentioned above, it is conceivable that the adverse health effects caused by MeHg in humans are due to oxidative damage. Therefore, studies which aim to evaluate the protective effects of food-compounds that can counteract the MeHg-induced oxidative damage in macromolecules, such as in DNA, are in need to have a better knowledge about the mechanisms that these compounds counteract the adverse effects induced by the metal and consequently may help to protect populations that are exposed chronically to MeHg.

## 5. Conclusions

The present study is the first which concerns the protective effects of the flavonoid CR against DNA-damage induced by exposure of MeHg* in vivo*. The results give further support about the fact that CR itself does not cause adverse health effects in mammals and indicate that flavonoid may protect against DNA-damage and disturbances in redox status induced by the metal compound.
